# Valorization of the Invasive Fish *Atherina boyeri* (Risso, 1810) as a Source of Protein Hydrolysates with Functional and Bioactive Properties

**DOI:** 10.3390/foods15020330

**Published:** 2026-01-16

**Authors:** Irem Ceren Kizilkoy, Sefik Tekle, Fatih Bozkurt, Hamza Goktas, Fahriye Seyma Ozcan, Mahmut Yilmaz, Osman Sagdic

**Affiliations:** 1 Department of Agricultural Biotechnology, Faculty of Agricultural, Kirsehir Ahi Evran University, Kirsehir 40100, Türkiye; myilmaz@ahievran.edu.tr; 2 Department of Food Engineering, Faculty of Engineering and Architecture, Kirsehir Ahi Evran University, Kirsehir 40100, Türkiye; 3 Department of Food Engineering, Faculty of Chemical and Metallurgical Engineering, Yildiz Technical University, Davutpasa Campus, Istanbul 34210, Türkiye; fbozkurt@yildiz.edu.tr (F.B.); osagdic@yildiz.edu.tr (O.S.); 4 Food Engineering Department, Engineering Faculty, Bolu Abant Izzet Baysal University, Bolu 14030, Türkiye; hamza.goktas@ibu.edu.tr; 5 TUBITAK Marmara Research Center, Life Sciences, Kocaeli 41470, Türkiye; seyma.bayraktar@tubitak.gov.tr

**Keywords:** invasive species valorization, sustainable protein sources, protein hydrolysates, antioxidant activity, techno-functional properties

## Abstract

The invasive fish *Atherina boyeri* constitutes an ecologically disruptive yet underexploited biomass with strong potential for transformation into value-added biofunctional ingredients. This study investigates the functional, antioxidant, and antimicrobial properties of protein hydrolysates that were produced from fish collected in the Hirfanlı and Yamula reservoirs using three commercial proteases (alcalase, bromelain, and flavourzyme). Bromelain produced the highest degree of hydrolysis, yielding higher proportions of low-molecular-weight peptides and greater radical-scavenging activity. Flavourzyme hydrolysates exhibited the most favorable emulsifying properties, Alcalase hydrolysates produced the highest foaming capacity and stability. All hydrolysates showed high absolute zeta-potential values across pH 3–9, demonstrating strong colloidal stability. Protein solubility remained above 80% across most pH levels, indicating extensive peptide release and improved compatibility with aqueous media. The Oil-binding capacity (2.78–3.75 mL/g) was consistent with reported values for marine hydrolysates. Antioxidant and antimicrobial evaluations revealed clear enzyme-dependent patterns, with Bromelain exhibiting the strongest DPPH activity and Alcalase and Flavourzyme showing the most pronounced inhibition of major foodborne pathogens. Additionally, all hydrolysates exhibited measurable ACE-inhibitory activity, with flavourzyme-derived peptides showing the highest inhibitory activity, underscoring their potential relevance for antihypertensive applications. These findings highlight the strategic valorization of *A. boyeri* through enzymatic hydrolysis, demonstrating its potential as a sustainable, clean-label functional ingredient source.

## 1. Introduction

The global fish industry represents a vital economic sector and a major source of high-quality protein for millions of people, particularly in developing countries. It is estimated that the livelihoods of nearly one billion individuals worldwide depend on fish production, processing, and trade [1]. Global fish production reached 185 million tons in 2022 and is projected to increase to 205 million tons by 2032; however, only 164.6 million tons are destined for direct human consumption [2]. A substantial fraction of aquatic biomass, therefore, remains underutilized, either diverted to low-value uses such as fishmeal and fish oil or generated as processing by-products, including skins, heads, viscera, livers, and bones, which may account for up to 60% of total fish biomass and pose environmental concerns if improperly managed [3]. While these non-edible resources are increasingly recognized as promising substrates for the production of value-added protein ingredients due to their high protein content [4], their availability is often constrained by seasonal processing and industrial demand. In this context, invasive fish species—frequently excluded from conventional food chains and considered ecologically problematic—represent an alternative and sustainable protein source. The valorization of such species offers a complementary strategy to by-product utilization, enabling the recovery of biofunctional compounds while simultaneously contributing to invasive species management and resource efficiency.

Protein hydrolysates, obtained through enzymatic cleavage of proteins into small peptides (2–20 amino acids), offer promising applications in both food and nutraceutical industries. Hydrolysis improves digestibility, enhances solubility, and imparts valuable functional properties, including emulsification, foaming, and gelling [5,6]. Compared with chemical or fermentation-based hydrolysis, enzymatic methods are particularly advantageous due to their high yields, nutritional preservation, and ability to generate bioactive peptides [7]. As a result, fish protein hydrolysates have attracted increasing interest as sustainable and value-added ingredients with health-promoting and technological benefits.

Aquatic invasive species are organisms introduced beyond their native range that successfully proliferate in new aquatic environments, where high feeding pressure, rapid population growth, and wide ecological tolerance drive ecosystem imbalance by reshaping food webs, displacing native fauna, and reducing the abundance of species of both ecological and commercial relevance [8]. While their uncontrolled proliferation is widely associated with biodiversity loss and ecosystem imbalance, the targeted valorization of invasive fish populations has recently been proposed as a sustainable management strategy that simultaneously mitigates ecological risk and enables the production of high-value food and nutraceutical ingredients [8,9,10]. *Atherina boyeri* (Risso, 1810), commonly known as the big-scale sand smelt or silverfish, is one such invasive species. Originally marine, this species has successfully colonized freshwater and brackish systems, aided by traits such as early sexual maturity, short lifespan, and extended spawning periods [11,12]. In Turkish inland waters, *A. boyeri* is particularly abundant, exerting predation pressure on zooplankton and competes with endemic and economically important fish, thereby threatening biodiversity and ecosystem balance [13].

Despite its ecological risks, *A. boyeri* also represents a protein-rich but underutilized biomass. In 2024, nearly 6680 tons were harvested in Türkiye [14], with most directed toward fishmeal or animal feed, and only limited quantities used in food products such as marinades [11], snacks [15], or processed items [16]. Harnessing this invasive species for the production of protein hydrolysates not only contributes to mitigating its ecological impact but also enables the generation of high-value, sustainable ingredients for food and nutraceutical applications.

Although a few studies have explored protein hydrolysates derived from invasive fish species such as silver carp (*Hypophthalmichthys molitrix*) [10,17], armoured catfish (*Pterygoplichthys disjunctivus*) [8], and red lionfish (*Pterois volitans*) [9], research on *Atherina boyeri* remains nonexistent. Therefore, this study sought to valorize *A. boyeri* through controlled enzymatic hydrolysis and systematically characterize the resulting protein hydrolysates with respect to their techno-functional and bioactive properties. To the best of our knowledge, this is the first comprehensive investigation of hydrolysates produced from this invasive species. By integrating invasive species management with enzyme-driven, clean-label protein hydrolysis that eliminates chemical additives and minimizes processing inputs, this study establishes a robust and sustainable valorization strategy for transforming *Atherina boyeri* into a high-value functional raw material for advanced food and nutraceutical applications.

## 2. Materials and Methods

### 2.1. Materials

In this study, three commercially available proteolytic enzymes—alcalase (≥0.75 Anson units/mL), bromelain (≥3 units/mg protein), and flavourzyme (500–1000 LAPU/g)—were employed due to their previously reported effectiveness in generating fish protein hydrolysates. Alcalase and bromelain were supplied by Sigma-Aldrich (St. Louis, MO, USA), while Flavourzyme was sourced from Novozymes (Bagsværd, Denmark). All analytical reagents, including phosphate-buffered saline (PBS) and sodium dodecyl sulfate (SDS), were of analytical grade and obtained from Sigma-Aldrich (St. Louis, MO, USA).

#### Raw Material

Silverfish (*Atherina boyeri* Risso, 1810), an invasive species inhabiting the Yamula and Hirfanlı Dam Lakes (Türkiye), was supplied by local fishing companies using conventional capture methods. Specimens were collected during March, April, and May (2025). Upon arrival at the laboratory, the fish, which had already been dead at the time of collection and had been kept under refrigerated conditions, were eviscerated using sterilized forceps, thoroughly washed to remove external impurities, and allowed to drain. After removal of the viscera, whole *Atherina boyeri* samples, including fillet, skin, and bones, were portioned into 500 g batches, vacuum-packed, and stored at −20 °C until further use. Prior to hydrolysate production, the frozen samples were thawed at 4 °C for 24 h and subsequently homogenized using a meat grinder (Arzum, Istanbul, Türkiye).

### 2.2. Preparation of Fish Protein Hydrolysates (FPH)

The minced *Atherina boyeri* samples were homogenized with distilled water at a solid-to-liquid ratio of 1:3 (*w*/*v*). The reaction mixtures were adjusted to the optimal pH and temperature conditions for each enzyme based on manufacturer’s specifications and previous studies: pH 8.0 at 60 °C for Alcalase and pH 7.0 at 50 °C for both bromelain and Flavourzyme. Adjustments were made using 1 M NaOH or HCl. Enzymatic hydrolysis was initiated by adding the enzyme at 1% (*w*/*w* protein), as determined by preliminary protein content analyses. Hydrolysis was carried out for 3 h, a duration optimized in preliminary trials in which longer incubations did not significantly alter the degree of hydrolysis. After completion of enzymatic hydrolysis, insoluble materials were removed by filtration, and the filtrates were clarified by centrifugation at 15,000× *g* for 20 min. The supernatants were heated at 90 °C for 20 min to inactivate residual proteolytic activity. Finally, the protein hydrolysates were freeze-dried and stored in powdered form until further analysis. In total, six fish protein hydrolysates were prepared: HSalc and YSalc using alcalase; HSbro and YSbro using bromelain; and HSfla and YSfla using flavourzyme.

### 2.3. Determination of Degree of Hydrolysis (DH, %)

The degree of hydrolysis (DH) was quantified by the pH-stat technique, following the procedure reported by Gbogouri et al. [18]. This method relies on maintaining the reaction mixture at constant pH during enzymatic hydrolysis by automatic titration, compensating for the release or consumption of protons as peptide bonds are cleaved. The volume of alkali required to sustain the target pH during hydrolysis was continuously monitored and subsequently applied in the calculation of DH. The degree of hydrolysis is defined as the ratio of hydrolyzed peptide bonds (h) to the total number of peptide bonds per protein equivalent (h_tot_). The calculation was performed according to the following equation:

(1) Degree of Hydrolysis (%) = (h × 100)/h_tot_ = (B × N_b_ × 100)/(α × Mp × h_tot_)

where

B = volume of base consumed (mL);

Nb = normality of the base (1 N);

α = average dissociation degree of the α-NH_2_ groups;

Mp = mass of protein (g L^−1^);

h_tot_ = total number of peptide bonds per gram of protein (meq/g protein).

### 2.4. Sodium Dodecyl Sulfate–Polyacrylamide Gel Electrophoresis (SDS–PAGE) Analysis

SDS–PAGE profiling was carried out based on the protocol of Bozkurt et al. [5] with minor adaptations. Resolving gels (7.5% and 20%) and a 4% stacking gel were cast for protein separation. Freeze-dried hydrolysates were combined with sample buffer (1:1, *v*/*v*) comprising 0.5 M Tris–HCl (pH 6.8), glycerol, 10% (*w*/*v*) SDS, 0.5% (*w*/*v*) bromophenol blue, and β-mercaptoethanol, followed by thermal denaturation at 100 °C for 5 min. Subsequently, 20 µL of each preparation was loaded into the gel wells, and electrophoresis was performed using a vertical system (Mini-PROTEAN^®^ Cell, Bio-Rad, Hercules, CA, USA) at a constant current of 20 mA per gel. Protein bands were visualized by staining with 0.1% (*w*/*v*) Coomassie Brilliant Blue R-250 in 40% methanol and 10% acetic acid; excess dye was removed by destaining with 40% ethanol and 10% acetic acid. Gel images were captured using a Gel Doc EZ imaging system (Bio-Rad, Marnes-la-Coquette, France).

### 2.5. Measurement of Surface Characteristics

#### 2.5.1. Zeta Potential

The zeta potential of fish protein hydrolysates was determined according to the method of Tekle [19] with slight modifications. A 10% (*w*/*v*) hydrolysate solution was prepared in distilled water and incubated in a water bath at 45 °C for 30 min to ensure complete dissolution (Memmert WNB45, Schwabach, Germany). Subsequently, 25 μL of the sample was mixed with 2 mL of phosphate-buffered saline (PBS, 0.01 M, pH 7.4). Measurements were carried out using a Zetasizer Nano ZSP (Malvern Instruments, Worcestershire, UK). Zeta potential values were recorded at pH 3, 5, 7, and 9; each condition measured at least in triplicate, and results were expressed as mean values.

#### 2.5.2. Determination of Active Sulfhydryl (SH) Content

The active sulfhydryl content of fish protein hydrolysates was measured using 5,5′-dithiobis-(2-nitrobenzoic acid) (DTNB), following the method of Gong et al. [20]. Briefly, 180 mg of protein was dissolved in 30 mL of Tris-glycine buffer (0.086 M Tris, 0.09 M glycine, 4 mM EDTA, pH 8.0) containing 8 M urea and mixed at room temperature for 30 min. The solution was centrifuged at 10,000× *g* for 10 min (Andreas Hettich GmbH & Co. KG, Tuttlingen, Germany), and the supernatant was collected. To determine the SH content, 4 mL of the diluted supernatant was added to 160 μL of DTNB solution (4 mg/mL in the same buffer) and the mixture was incubated for 15 min. The absorbance of the mixture was then recorded at 412 nm using a spectrophotometer (Shimadzu UV-1800, Tokyo, Japan). A reagent blank (buffer + DTNB) was used as the control. The active sulfhydryl content was calculated according to the following equation:

(2) Active SH groups (μmol/g) = 73.53 × A_412_/Sample concentration (mg/mL)

### 2.6. Amino Acid Composition

The amino acid composition of the samples was determined following the procedure described by Özcan [21], with minor modifications. Briefly, the samples were hydrolyzed overnight at 110 °C with 6 M HCl. After hydrolysis, the solutions were filtered through a 0.45 µm membrane and evaporated to dryness in glass vials under a nitrogen atmosphere. Subsequently, derivatization was carried out by adding phenyl isocyanate, followed by incubation at 40 °C for 30 min. The mixture was again dried under nitrogen and washed with acetonitrile (ACN). After a second drying step under nitrogen, the residues were reconstituted in 0.02 M ammonium acetate. The solution was sonicated in a water bath for 5 min, filtered (0.45 µm), and analyzed by HPLC–UV (LC-20A, Shimadzu, Tokyo, Japan). All analyses were performed in triplicate.

### 2.7. Fourier Transform Infrared (FTIR) Spectroscopy

Fourier transform infrared (FTIR) spectroscopy was employed to examine the structural characteristics and functional groups of the fish protein hydrolysates, with particular emphasis on the amide I, II, III, A, and B regions, in accordance with the methodology reported by Cebi et al. [22]. Hydrolysate solutions (10% *w*/*v*) were prepared in distilled water and heated at 45 °C for 30 min in a thermostated water bath (Memmert WNB45, Schwabach, Germany) to ensure complete dissolution and homogeneity. ATR–FTIR measurements were carried out using a Bruker Tensor 27 spectrometer (Ettlingen, Germany), operating at a spectral resolution of 4 cm^−1^ with 16 accumulated scans per measurement. Spectra were acquired over the mid-infrared range of 4000–600 cm^−1^. Each sample was analyzed in triplicate under identical experimental conditions, and mean spectra were used for subsequent evaluation. Prior to each analysis, the ATR crystal was thoroughly cleaned with distilled water followed by absolute ethanol to prevent cross-contamination.

### 2.8. Color Measurement

The color attributes of the fish protein hydrolysates were measured using a colorimeter (CR-100, Konica Minolta, Tokyo, Japan). The CIE Lab* color coordinates were recorded, where L* denotes lightness on a scale from 0 (black) to 100 (white), a* describes the chromatic axis from red (+) to green (−), and b* represents the axis from yellow (+) to blue (−). The total color difference (ΔE*) was calculated using the equation reported by Tekle et al. [23]:

(3) ΔE* = [(ΔL*)^2^ + (Δa*)^2^ + (Δb*)^2^]^1/2^

where ΔL*, Δa*, and Δb* represent the differences in the L*, a*, and b* values among the fish protein hydrolysates. Color measurements for each sample were conducted in three independent replicates, and the averaged values were used for subsequent analysis.

### 2.9. Functional Properties of FPH

#### 2.9.1. Protein Solubility

The protein solubility of fish hydrolysates was determined following the procedure described by the American Oil Chemists’ Society [24]. Hydrolysate samples were dispersed in distilled water, and the pH of the resulting solutions was adjusted to 3, 5, 7, and 9 using 0.5 N NaOH or 0.5 N HCl while stirring continuously for 45 min. The mixtures were then centrifuged at 2800× *g* for 30 min (Andreas Hettich GmbH & Co. KG, Tuttlingen, Germany). The nitrogen content of 15 mL aliquots of the supernatants was determined using the Kjeldahl method. Protein solubility was expressed as follows:

(4) Protein solubility (%) = (Protein content in the supernatant/Total protein content) × 100

#### 2.9.2. Emulsifying Properties

The emulsifying activity index (EAI) and emulsion stability index (ESI) of the fish protein hydrolysates were determined as described in previous studies with slight modifications [19]. Briefly, 90 mg of hydrolysate was dispersed in 9 mL of phosphate-buffered saline (PBS) and homogenized at high speed. Subsequently, sunflower oil (3 mL) was incorporated into the mixture, which was then subjected to a second homogenization step at 18,000× *g* for 1 min. Aliquots (50 μL) of the emulsion were collected immediately (0 min) and after 10 min; each aliquot was diluted to 5 mL with 0.1% (*w*/*v*) sodium dodecyl sulfate (SDS) solution. The absorbance of the diluted emulsions was measured at 500 nm using a spectrophotometer.

EAI and ESI values were calculated using the following equations:

(5) EAI (m^2^/g) = (2 × 2.303 × 100 × A)/(c × 0.25 × 10,000)

A = absorbance at 500 nm;

c = protein concentration (g/mL).

(6) ESI (dk) = (A_0_ × 10)/(A_0_ − A_10_)

A_0_ = absorbance at 0 min;

A_10_ = absorbance at 10 min.

#### 2.9.3. Foaming Capacity and Stability

Foaming capacity (FC) and foam stability (FS) were determined according to the method of Tekle [19] with slight modifications. A hydrolysate dispersion (0.5% *w*/*v*) was prepared in distilled water, and a 10 mL aliquot was homogenized at 14,000× *g* for 1 min at ambient temperature using a VELP OV5 homogenizer (Usmate, Italy). Foam volume was measured immediately after homogenization (0 min) and again after 10 min. Foaming capacity was calculated based on the initial foam expansion, whereas foam stability was defined as the proportion of foam volume remaining after 10 min. Foam expansion was determined using the following equation:

(7) Foam expansion (%) = [(A − B) /B] × 100

where

A = foam volume at a given time (mL);

B = initial liquid volume before homogenization (mL).

#### 2.9.4. Oil Binding Capacity

Oil binding capacity (OBC) was assessed following the procedure of Tekle [19] with minor adjustments. Briefly, 50 mg of each hydrolysate were placed into pre-weighed centrifuge tubes and mixed with 1.5 mL of sunflower oil. The mixtures were incubated at room temperature for 1 h and vortexed for 5 s at 15 min intervals using a VELP ZX3 vortex mixer (Usmate, Italy). After incubation, samples were centrifuged at 5000× *g* for 15 min (Andreas Hettich GmbH & Co. KG, Tuttlingen, Germany), and the oil-containing supernatant oil was carefully decanted. Control tubes containing only sunflower oil were processed in parallel. OBC was expressed as milliliters of oil retained per gram of protein.

### 2.10. Bioactive Properties of FPH

#### 2.10.1. DPPH Radical Scavenging Activity

The DPPH radical scavenging activity of fish protein hydrolysates was evaluated according to the method of Dara et al. [25], with slight modifications. Hydrolysate solutions were prepared at concentrations of 0.75, 1.5, and 7.5 mg/mL in distilled water. An aliquot of 1.5 mL of hydrolysate solution was mixed with 1.5 mL of 0.2 mM DPPH solution in ethanol. The mixture was vortexed (Velp ZX3 Vortex Mixer, Usmate (MB), Italy) and incubated in the dark at room temperature for 20 min. Control samples were prepared using distilled water instead of hydrolysate solution. The absorbance was measured at 517 nm using a spectrophotometer (Shimadzu UV-1800, Tokyo, Japan).

The radical scavenging activity was calculated as follows:

(8) DPPH Scavenging Activity (%) = (A_control_ − A_sample_/A_control_ ) × 100

where A_control_ is the absorbance of the control and A_sample_ is the absorbance of the hydrolysate solution.

#### 2.10.2. Determination of Antimicrobial Activity

The lyophilized silverfish protein hydrolysates were dissolved in sterile distilled water to produce dilutions of 1:1, 1:2, and 1:4 (mg/μL). Pathogenic strains including *Bacillus cereus* BC 6830, *Salmonella typhimurium* RSSK 95091, *Escherichia coli* ATCC 25922, and *Staphylococcus aureus* ATCC 25923, were inoculated (1% *v*/*v*) into nutrient agar medium tempered to the pouring temperature. The agar well diffusion method was employed, and three wells were aseptically punched into each plate. Subsequently, 60 μL of hydrolysate solutions of varying concentrations were introduced into the wells, and sterile distilled water served as the negative control. Plates were incubated for 18 h, and the inhibition zones were measured and expressed in millimeters [26].

#### 2.10.3. Angiotensin-I Converting Enzyme (ACE) Inhibitory Activity

The ACE inhibitory activity of silverfish protein hydrolysates was evaluated following the method described by Yu et al. [27], with slight modifications. The substrate HHL was dissolved at 5 mM in 0.1 M sodium borate buffer (pH 8.3) containing 0.3 M NaCl. The assay was performed by mixing 100 μL of substrate solution with 25 μL of inhibitor solution (or with 25 μL of borate buffer for the control). After 10 min of incubation at 37 °C, 10 μL of ACE solution (100 mU/mL) was added and the sample (1 mg/mL) was further incubated at 37 °C for 30 min with continuous agitation at 450 rpm. The reaction was stopped by the addition of 100 μL of 1 M HCl and the solution was filtered through a 0.45-μm nylon syringe filter before being analyzed by reversed-phase HPLC. The HPLC analysis was performed on a C18 column (250 × 4.6 mm i.d., particle size 5 μm) using a Varian Shimadzu chromatographic system and analytes were detected at the wavelength of λ = 228 nm. The column was eluted at a flow rate of 0.5 mL min^−1^. The isocratic mobile phase consisted of 25% acetonitrile in deionized water (*v*/*v*) with 0.5% TFA. It was filtered through 0.45 μm cellulose filters and degassed by ultrasonication for 30 min. A 10 μL aliquot of the reaction mixture was analyzed by reverse-phase HPLC. Hippuryl-l-histidyl- l-leucine and hippuric acid were detected at 228 nm when the mobile phase flow rate was maintained at 0.5 mL/min. The ACE inhibition percentage was calculated using the following equation:

(9) ACE inhibition (%) = (A − B/A − C ) × 100

where A is the absorbance of the control (without sample), B is the absorbance of the reaction mixture with the sample, and C is the blank absorbance (without ACE and sample).

### 2.11. Statistical Analysis

All analyses were conducted in triplicate, and results were reported as mean ± standard deviation. Statistical evaluation was performed using one-way ANOVA in JMP Pro 18 (SAS Institute Inc., Cary, NC, USA), followed by Tukey’s HSD post hoc test to identify significant differences, with *p* < 0.05 was considered statistically significant.

## 3. Results and Discussion

### 3.1. Determination of Degree of Hydrolysis (DH, %)

**Figure 1:**
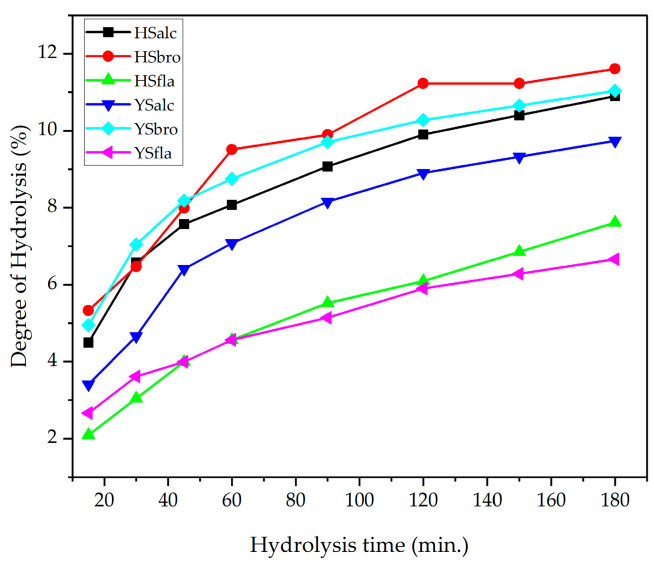
Degree of hydrolysis of silverfish protein hydrolysates. HSalc: Hirfanlı silverfish alcalase hydrolysate, HSbro: Hirfanlı silverfish bromelain hydrolysate, HSfla: Hirfanlı silverfish flavourzyme hydrolysate, YSalc: Yamula silverfish alcalase hydrolysate, YSbro: Yamula silverfish bromelain hydrolysate, YSfla: Yamula silverfish flavourzyme hydrolysate.

The degree of hydrolysis (DH) is a key indicator of proteolytic reaction progress and reflects the proportion of cleaved peptide bonds during enzymatic hydrolysis. The DH values of the six prepared hydrolysates (HSalc, HSbro, HSfla, YSalc, YSbro, YSfla) are shown in Figure 1. After 180 min of hydrolysis, the DH ranged from 6.66% to 11.60%, with the highest values obtained in bromelain-treated samples (HSbro: 11.60%; YSbro: 11.04%), followed by alcalase-treated samples (HSalc: 10.90%; YSalc: 9.74%). Flavourzyme hydrolysates exhibited the lowest DH (HSfla: 7.61%; YSfla: 6.66%). All enzyme treatments displayed a sharp increase in DH within the first 60 min, followed by a gradual slowdown. This pattern is typical of enzymatic hydrolysis and is associated with the reduction in available substrate and the accumulation of reaction products that may interfere with enzyme activity. Similar hydrolysis kinetics have been reported for fish proteins, including Pacific whiting hydrolyzed using alcalase [28]. The enzyme-specific differences were evident. Bromelain and alcalase, both of which possess broad proteolytic specificity, produced significantly higher DH values than flavourzyme, whose predominantly exopeptidase activity limits its ability to cleave peptide bonds rapidly. These findings are consistent with previous studies demonstrating the strong hydrolytic capacity of bromelain and alcalase in fish protein systems [23,29,30]. Overall, the data confirm that the extent of protein hydrolysis is primarily governed by enzyme type, with bromelain and alcalase yielding more extensive peptide bond cleavage than flavourzyme (Figure 1).

### 3.2. Sodium Dodecyl Sulfate–Polyacrylamide Gel Electrophoresis (SDS–PAGE) Analysis

**Figure 2:**
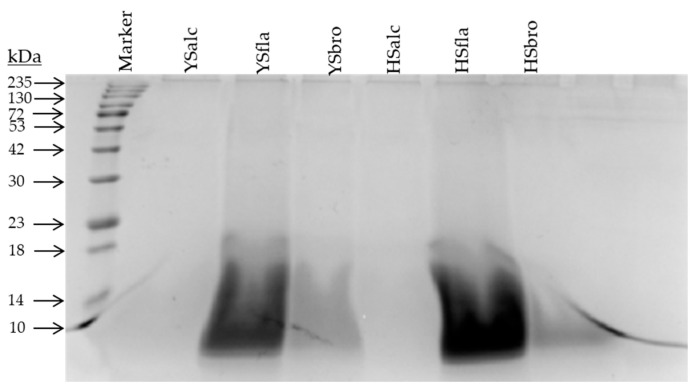
The SDS-PAGE patterns of silverfish protein hydrolysates. YSalc: Yamula silverfish alcalase hydrolysate, YSfla: Yamula silverfish flavourzyme hydrolysate, YSbro: Yamula silverfish bromelain hydrolysate, HSalc: Hirfanlı silverfish alcalase hydrolysate, HSfla: Hirfanlı silverfish flavourzyme hydrolysate, HSbro: Hirfanlı silverfish bromelain hydrolysate.

The molecular weight distribution of the silverfish protein hydrolysates produced using three different proteolytic enzymes (alcalase, bromelain, and flavourzyme) was evaluated by SDS-PAGE (Figure 2). Clear enzyme-dependent differences were observed, indicating distinct hydrolysis intensities among treatments. Hydrolysates generated with alcalase exhibited almost no discernible protein bands and showed an extensive smear across the low–molecular weight region. This pattern reflects severe proteolytic degradation, indicating that most proteins were cleaved into short peptides. Such extensive fragmentation is consistent with the strong endoproteolytic activity commonly attributed to alcalase. The bromelain hydrolysate displayed a moderate smear with noticeably weakened band intensities, suggesting an intermediate degree of hydrolysis. Bromelain possesses both endo- and exoproteolytic activities, which accounts for the partial breakdown of high- and medium–molecular weight proteins while still retaining some residual polypeptide structure. Accordingly, the SDS-PAGE profile revealed partial fragmentation, though the degradation was less pronounced than that observed in the alcalase treatment. In contrast, hydrolysates produced with flavourzyme retained clearer, more distinct protein bands, particularly near the molecular-weight marker. A reduced smear and more defined banding pattern indicate that flavourzyme produced in the lowest degree of hydrolysis among the enzymes tested. This observation aligns with flavourzyme’s predominant exopeptidase activity, which generally produces larger peptides and slows the fragmentation of intact proteins. The presence of more intact polypeptide chains is consistent with the lower hydrolysis degree measured analytically. Collectively, the SDS-PAGE results confirm an enzyme-dependent hydrolysis hierarchy: Alcalase > Bromelain > Flavourzyme, with respect to protein degradation and peptide size reduction. Similar patterns have been reported for hydrolysates derived from red lionfish, yellow stripe trevally and silver carp, reinforcing the consistency of the observed trend [8,10,31].

### 3.3. Measurement of Surface Characteristics

#### 3.3.1. Zeta Potential

**Figure 3:**
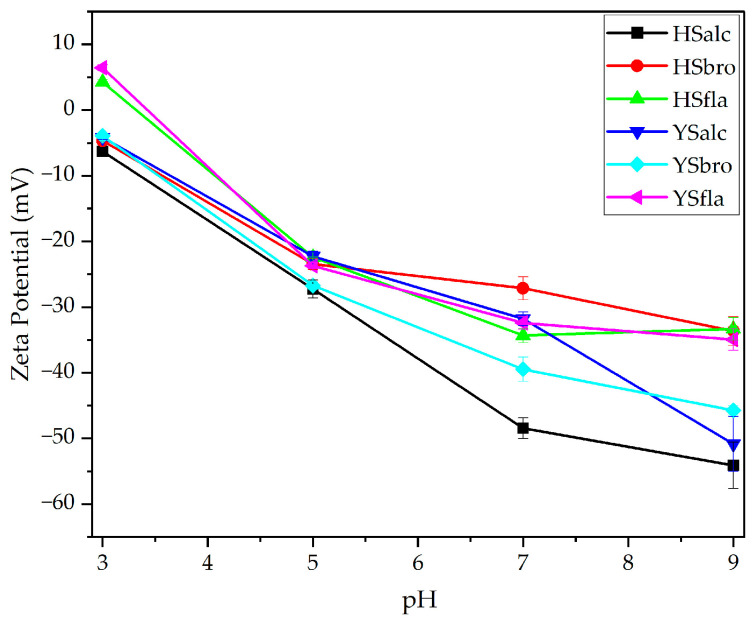
Zeta potentials of silverfish protein hydrolysates. Error bars denote standard deviation (*n* = 3). HSalc: Hirfanlı silverfish alcalase hydrolysate, HSbro: Hirfanlı silverfish bromelain hydrolysate, HSfla: Hirfanlı silverfish flavourzyme hydrolysate, YSalc: Yamula silverfish alcalase hydrolysate, YSbro: Yamula silverfish bromelain hydrolysate, YSfla: Yamula silverfish flavourzyme hydrolysate.

Zeta potential reflects the electrostatic charge balance and colloidal stability of protein hydrolysates, serving as an indicator of their ability to remain dispersed in solution without aggregation [32]. The zeta potential values of silverfish protein hydrolysates exhibited a clear pH-dependent behavior, reflecting alterations in net surface charge and colloidal stability across the acidic–alkaline range (Figure 3). At pH 9, all hydrolysates exhibited strongly negative charges; however, alcalase hydrolysates (HSalc and YSalc) exhibited significantly more negative zeta potentials than both bromelain and flavourzyme hydrolysates (*p* < 0.05). Among all samples, HSalc exhibited the most pronounced electrostatic repulsion (−54.1 mV), indicating superior colloidal stability under alkaline conditions [33]. At neutral pH, a general reduction in charge magnitude was observed. HSalc remained significantly more negative than the corresponding Yamula hydrolysates and all flavourzyme-treated samples (*p* < 0.05), suggesting that alcalase digestion preserved surface-active charged groups more effectively. At pH 5, all hydrolysates converged into a narrower range (−22.3 to −27.3 mV), consistent with approaching the isoelectric region. Despite this overall decline, HSalc and YSbro exhibited slightly more negative values, which differed statistically from those of the other enzyme groups (*p* < 0.05). This pattern indicates that partial retention of acidic side chains may contribute to residual charge stability. Pronounced differences emerged at pH 3, where flavourzyme hydrolysates (HSfla and YSfla) shifted into the positive region (+4.24 and +6.44 mV, respectively), while alcalase and bromelain hydrolysates remained slightly negative (−6.28 to −3.92 mV). The positive shift observed in flavourzyme samples was statistically significant compared with all other hydrolysates (*p* < 0.05) and suggests extensive exposure of protonatable amino groups due to enzyme-specific cleavage patterns. The zeta potential of all hydrolysates became increasingly negative with rising pH, reflecting the progressive deprotonation of surface-exposed functional groups. Similar pH-dependent shifts have previously been reported for alcalase- and protamex-derived hydrolysates from *Micropogonias furnieri* and *Paralonchurus brasiliensis*, supporting the consistency of this electrokinetic behavior across fish protein hydrolysate systems [33]. Overall, statistical analysis indicated that enzyme type had a significant main effect on zeta potential across all pH levels (*p* < 0.05). The ordering Alcalase > Bromelain > Flavourzyme, with respect to the magnitude of negative charge, was maintained across the pH range 3–9. These findings demonstrate that alcalase hydrolysates possess the highest electrostatic stability, whereas flavourzyme hydrolysates are more susceptible to charge neutralization at low pH, which may, in turn, influence their solubility, aggregation behavior, and functional applications in acidic food systems.

#### 3.3.2. Determination of Active Sulfhydryl (SH) Content

**Figure 4:**
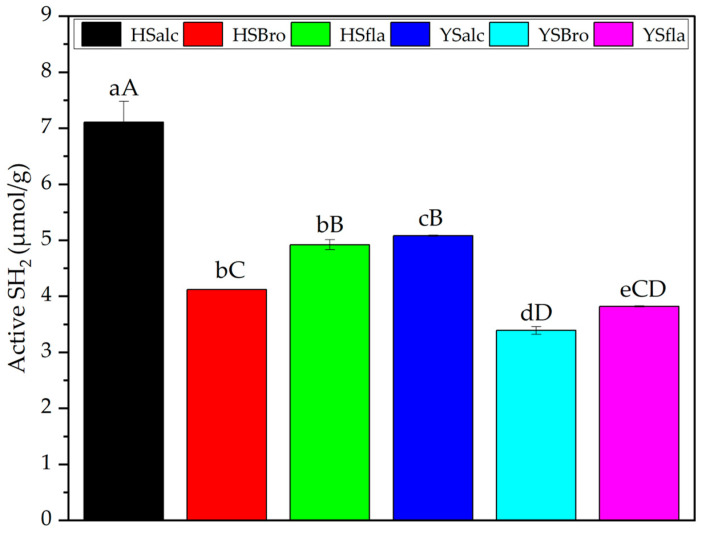
Active sulfhydryl groups (SH_2_) of silverfish protein hydrolysates. Results are reported as the mean and associated standard deviation of three independent measurements; error bars represent the corresponding variability (n = 3). Values with different lowercase letters differ significantly within each reservoir, whereas different uppercase letters indicate significant differences among all samples (*p* < 0.05). HSalc: Hirfanlı silverfish alcalase hydrolysate, HSbro: Hirfanlı silverfish bromelain hydrolysate, HSfla: Hirfanlı silverfish flavourzyme hydrolysate, YSalc: Yamula silverfish alcalase hydrolysate, YSbro: Yamula silverfish bromelain hydrolysate, YSfla: Yamula silverfish flavourzyme hydrolysate.

The SH_2_ activities of hydrolysates produced from Hirfanlı silverfish (HS) and Yamula silverfish (YS) using alcalase, bromelain, and flavourzyme are presented in Figure 4. Among HS hydrolysates, HSalc exhibited the highest SH_2_ activity (7.11 ± 0.37 μmol/g), followed by HSfla (4.92 ± 0.09 μmol/g). The lowest activity was observed in HSbro (4.12 ± 0 μmol/g). A similar trend was observed for YS hydrolysates, where YSalc showed the highest activity (5.08 ± 0.01 μmol/g) and YSbro displayed the lowest (3.39 ± 0.07 μmol/g). YSfla demonstrated intermediate activity (3.82 ± 0.009 μmol/g) for this species. These differences likely arise from the distinct catalytic specificities and hydrolysis patterns of the enzymes used. Alcalase consistently produced the highest SH_2_ levels in both species, suggesting its superior ability to expose or retain active thiol groups during hydrolysis. In contrast, bromelain-generated hydrolysates exhibited the lowest SH_2_ activity, which may reflect reduced proteolytic efficiency or partial enzyme inactivation during processing. Flavourzyme yielded intermediate results, consistent with its mixed endo- and exopeptidase activity. Species-related effects were also apparent. HS hydrolysates generally exhibited higher SH_2_ activity than YS samples, particularly in alcalase-treated fractions, indicating inherent differences in protein composition or susceptibility to enzymatic cleavage between silverfish populations. Overall, HSalc was the hydrolysate with the highest SH_2_ activity, whereas YSbro exhibited the lowest SH_2_ activity. These findings highlight the combined influence of enzyme type and species origin on SH_2_ activity, in agreement with previous reports showing that both factors significantly modulate functional and biochemical characteristics of fish-derived hydrolysates [34].

### 3.4. Amino Acid Composition

The amino acid composition of the hydrolysates produced using alcalase, bromelain, and flavourzyme is shown in Table 1. According to Klompong et al. [31], variations in amino acid profiles are largely attributable to differences in hydrolysis parameters, including the specific protease employed as well as the pH and temperature conditions applied during the enzymatic reaction. Lysine and glutamic acid were the dominant amino acids across all samples, with lysine showing the highest concentrations (10,776–12,207.5 mg/kg), followed by glutamic acid (9477–11,526.5 mg/kg) and aspartic acid (5410–7035 mg/kg). This distribution is characteristic of freshwater fish proteins and indicates efficient cleavage of glutamate- and lysine-rich regions during hydrolysis [10]. In addition, the presence of glycine, proline, and alanine supports previous reports that these residues originate primarily from collagenous structures in the skin of *Atherina boyeri* [8]. Bromelain-treated hydrolysates generally exhibited higher levels of major amino acids, including glutamic acid, glycine, leucine, and alanine, suggesting broader proteolytic specificity and more extensive protein breakdown compared with alcalase and flavourzyme. Flavourzyme samples showed comparatively lower values for several amino acids, consistent with its predominant exopeptidase activity and slower cleavage of internal peptide bonds. Hydrophobic amino acids (e.g., leucine, isoleucine, valine, and alanine) also varied among treatments, with bromelain hydrolysates showing the highest levels, reflecting enhanced degradation of hydrophobic core regions—an observation relevant to functional properties such as antioxidant and emulsifying activities [17]. Differences between Hirfanlı and Yamula samples were generally minor, although YSbro exhibited some of the highest total amino acid levels, suggesting that population-specific biochemical factors may affect substrate susceptibility. Overall, the hydrolysis treatments effectively released both polar and hydrophobic residues, with bromelain yielding the greatest release of amino acid.

### 3.5. Fourier Transform Infrared (FTIR) Spectroscopy

**Figure 5:**
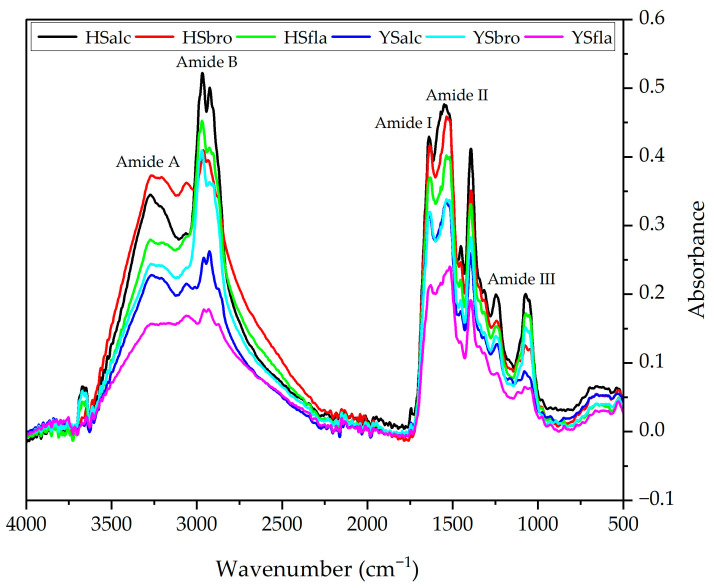
FTIR of silverfish protein hydrolysates. HSalc: Hirfanlı silverfish alcalase hydrolysate, HSbro: Hirfanlı silverfish bromelain hydrolysate, HSfla: Hirfanlı silverfish flavourzyme hydrolysate, YSalc: Yamula silverfish alcalase hydrolysate, YSbro: Yamula silverfish bromelain hydrolysate, YSfla: Yamula silverfish flavourzyme hydrolysate.

FTIR spectroscopy was used to assess structural features and functional group vibrations of the silverfish protein hydrolysates (Figure 5). The spectra of all six hydrolysates displayed the characteristic protein bands, including Amide I, II, III, A, and B, confirming the preservation of fundamental peptide backbone structures following enzymatic hydrolysis. The Amide I region, which is highly sensitive to secondary structure alterations, appeared at 1628–1638 cm^−1^ across all treatments, consistent with previously reported values for fish-derived hydrolysates. Amide II bands were detected between 1516 and 1550 cm^−1^, indicating N–H bending coupled with C–N stretching; minor shifts toward lower wavenumbers suggest increased hydrogen bonding. The Amide III band (1240–1393 cm^−1^) corresponded to a combination of C–N stretching and N–H deformation, reflecting backbone conformational features similar to those reported for other marine hydrolysates. Amide A peaks were observed at 3060–3271 cm^−1^, indicating modifications in N–H stretching vibrations associated with changes in the peptide environment and variations in hydrolysis intensity among enzymes. The Amide B region (2925–2969 cm^−1^) exhibited characteristic asymmetric C–H stretching vibrations and features attributable to secondary amines. Collectively, the FTIR patterns confirm that enzymatic hydrolysis did not substantially disrupt the primary protein structure but induced subtle alterations in hydrogen bonding, side-chain interactions, and local conformations, which are attributable to enzyme-specific cleavage behavior.

### 3.6. Color Measurement

Color attributes of the silverfish hydrolysates were strongly influenced by enzyme type, whereas location-based differences were comparatively minor (Table 2). Among all treatments, YSbro displayed the highest L* value, indicating the brightest appearance, and was significantly lighter than HSfla (*p* < 0.05). In contrast, HSalc, HSbro, YSalc, and YSfla showed statistically comparable lightness (*p* > 0.05), suggesting that bromelain exerted a more pronounced effect on color brightness at the Yamula site. For the a* coordinate, YSalc shifted most prominently toward the green axis and differed significantly from HSbro and YSfla (*p* < 0.05). The remaining samples—HSalc, HSfla, and YSfla—exhibited no significant differences (*p* > 0.05), but showed enzyme-dependent yet site-sensitive changes in red–green coloration. The b* values demonstrated greater variability, with HSalc showing the highest yellowness, significantly exceeding HSfla (*p* < 0.05). However, HSalc–HSbro and YSalc–YSfla pairs did not differ significantly, implying that alcalase-induced increases in b* were more evident only in specific fish batches. Importantly, ΔE values remained low and statistically indistinguishable among enzymes (ΔEalc: 1.94 ± 0.49ᵃ, ΔEbro: 2.11 ± 0.49ᵃ, ΔEfla: 3.15 ± 0.74ᵃ; *p* > 0.05), indicating that although individual color coordinates varied, overall perceived color differences were minimal. These findings are consistent with previous reports for tuna protein hydrolysates exhibiting a light creamy-yellow hue [35]. The subtle shifts observed here likely arise from differences in peptide release patterns, chromophoric amino acid exposure, and limited Maillard-type reactions during enzymatic hydrolysis [23]. Overall, the results demonstrate that enzyme specificity modulates the optical characteristics of the hydrolysates; however, the perceived color variation remains relatively small, which supports their potential suitability as functional food ingredients where color uniformity is desirable.

### 3.7. Functional Properties of FPH

#### 3.7.1. Protein Solubility

**Figure 6:**
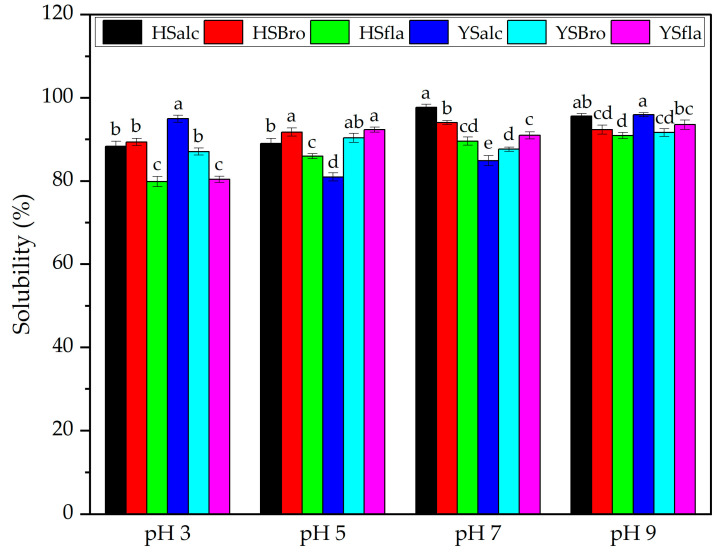
Protein solubility of silverfish protein hydrolysates. Results are reported as the mean of three independent measurements with associated standard deviations; error bars represent the corresponding variability (n = 3). Means labeled with different lowercase letters indicate statistically significant differences among all samples (*p* < 0.05). HSalc: Hirfanlı silverfish alcalase hydrolysate, HSbro: Hirfanlı silverfish bromelain hydrolysate, HSfla: Hirfanlı silverfish flavourzyme hydrolysate, YSalc: Yamula silverfish alcalase hydrolysate, YSbro: Yamula silverfish bromelain hydrolysate, YSfla: Yamula silverfish flavourzyme hydrolysate.

Protein solubility is one of the most critical functional indicators of enzymatically produced hydrolysates, as it directly influences their applicability in food formulations, particularly in emulsification, foaming, and dispersibility [36]. The solubility values of the silverfish hydrolysates at pH 3, 5, 7, and 9 are shown in Figure 6. Overall, all samples exhibited high solubility across the evaluated pH range, with values between 79.82% and 97.69%. Consistent with the general behavior of protein hydrolysates, solubility was lowest at acidic pH and increased progressively toward neutral and alkaline conditions, reflecting reduced aggregation as pH moved away from the isoelectric point and enhanced electrostatic repulsion at higher pH levels [37]. Across enzymatic treatments, alcalase-derived fractions (HSalc and YSalc) exhibited the highest solubility values under most pH conditions, reaching up to 97.69% at pH 9, which was significantly higher than those of flavourzyme-derived hydrolysates (*p* < 0.05). Comparable trends have been reported for hydrolysates generated from surimi processing by-products of silver carp using Alcalase and Protamex, in which enzymatic treatment similarly influenced the resulting physicochemical characteristics [32]. Bromelain hydrolysates (HSbro and YSbro) also exhibited high solubility, generally exceeding those of flavourzyme treatments, particularly at pH 3 and 5. HSfla and YSfla consistently showed the lowest values among their respective groups, which may be attributed to flavourzyme’s exopeptidase-dominant activity, resulting in a broader mixture of peptide lengths with partially reduced water affinity. These enzyme-dependent differences were statistically significant at all pH levels (*p* < 0.05). When comparing hydrolysates from the two sampling regions, no significant differences were detected between HS and YS samples at any pH (*p* > 0.05), suggesting that the origin of the raw material exerted a minimal influence on solubility compared with the enzyme used. This finding is consistent with previous studies, which report that fish protein hydrolysates—regardless of species—typically exhibit solubility values above 80% because of the formation of short, hydrophilic peptides [32,38]. The high solubility observed in this study indicates extensive peptide bond cleavage and exposure of polar functional groups, both of which facilitate strong interactions with water molecules and prevent aggregation [37]. Overall, the solubility behavior of the hydrolysates demonstrates that enzymatic hydrolysis markedly enhances protein–water interactions. The superior solubility of alcalase-derived hydrolysates highlights their potential suitability for applications requiring high dispersion stability, whereas the relatively low solubility of flavourzyme products suggests a more limited functional versatility depending on formulation needs.

#### 3.7.2. Emulsifying Properties

**Figure 7:**
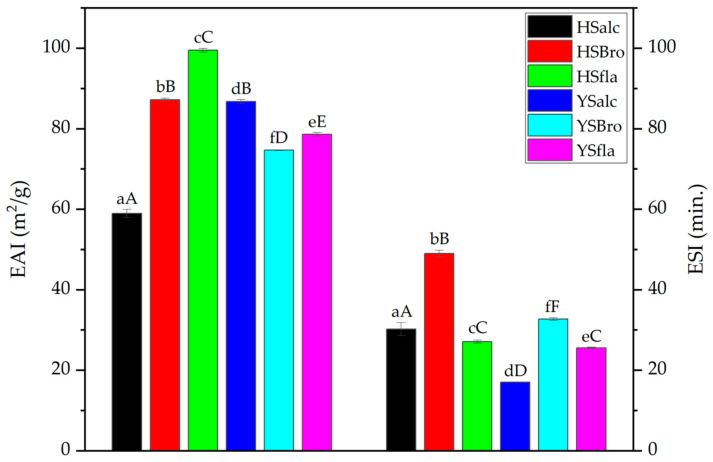
EAI and ESI of silverfish protein hydrolysates. Results are reported as the mean of three independent measurements with associated standard deviations; error bars represent the corresponding variability (n = 3). Values sharing different lowercase letters differ significantly within each reservoir, whereas different uppercase letters indicate significant differences among all samples (*p* < 0.05). HSalc: Hirfanlı silverfish alcalase hydrolysate, HSbro: Hirfanlı silverfish bromelain hydrolysate, HSfla: Hirfanlı silverfish flavourzyme hydrolysate, YSalc: Yamula silverfish alcalase hydrolysate, YSbro: Yamula silverfish bromelain hydrolysate, YSfla: Yamula silverfish flavourzyme hydrolysate. EAI: emulsifying activity index ESI: Emulsion stability index.

The emulsifying activity index (EAI) of the silverfish protein hydrolysates is shown in Figure 7. Among the hydrolysates, HSfla exhibited the highest EAI, whereas HSalc presented the lowest value. Since HSfla had the lowest degree of hydrolysis, this result is consistent with the principle that moderately hydrolyzed proteins—containing peptides of optimal chain length—adsorb more effectively at the oil–water interface [18]. Peptides generated during enzymatic hydrolysis interact at the interface through both hydrophilic and hydrophobic residues, forming a stabilizing interfacial film that prevents droplet coalescence [39]. The markedly lower EAI of HSalc may be attributed to extensive proteolysis by alcalase, resulting in short peptides with limited interfacial film–forming capacity. This mechanism is consistent with the explanation by Opazo-Navarrete et al. [40], who reported that excessive hydrolysis may lead to uneven peptide distribution at the interface, weakening emulsification performance. In addition, EAI values differed significantly between hydrolysates obtained from Hirfanlı and Yamula Lakes (*p* < 0.001), suggesting that raw material composition—such as muscle protein profile or inherent lipid content—also influences emulsifying behavior. Overall, the present findings indicate that moderate hydrolysis enhances EAI through an optimal amphiphilic balance of peptides, while over-hydrolysis limits interfacial adsorption efficiency.

The emulsion stability index (ESI) values of the silverfish protein hydrolysates are shown in Figure 7. Bromelain-derived hydrolysates (HSbro and YSbro) showed the highest stability among all samples, whereas YSfla presented the lowest ESI value. These findings agree with Elavarasan et al. [30], who demonstrated that bromelain-generated hydrolysates from *Catla catla* exhibited superior emulsion stability compared with those produced with alcalase and flavourzyme. The enhanced stability in bromelain hydrolysates can be attributed to the amphiphilic character of peptides formed during hydrolysis, which adsorb strongly to the oil–water interface and retard droplet aggregation [37]. Overall, the results suggest that bromelain produces peptide structures particularly well-suited for interfacial stabilization, whereas flavourzyme hydrolysis—especially in Yamula samples—yields peptides with insufficient ability to sustain long-term emulsion stability.

#### 3.7.3. Foaming Capacity and Stability

**Figure 8:**
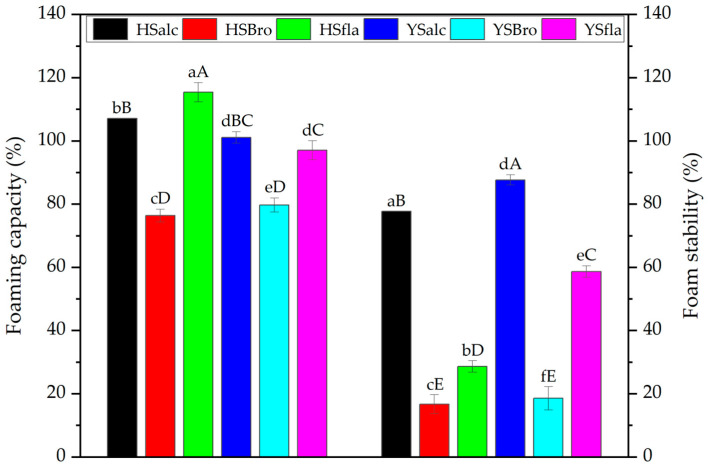
FC and FS of silverfish protein hydrolysates. Results are reported as the mean of three independent measurements with associated standard deviations; error bars represent the corresponding variability (n = 3). Values sharing different lowercase letters differ significantly within each reservoir, whereas different uppercase letters indicate significant differences among all samples (*p* < 0.05). HSalc: Hirfanlı silverfish alcalase hydrolysate, HSbro: Hirfanlı silverfish bromelain hydrolysate, HSfla: Hirfanlı silverfish flavourzyme hydrolysate, YSalc: Yamula silverfish alcalase hydrolysate, YSbro: Yamula silverfish bromelain hydrolysate, YSfla: Yamula silverfish flavourzyme hydrolysate. FC: Foam capacity, FS: Foam stability.

The foaming capacity (FC) and foam stability (FS) values of the silverfish protein hydrolysates are presented in Figure 8. FC varied significantly among enzyme treatments within each sampling site (*p* < 0.05). The highest FC was observed in the HSalc hydrolysate, whereas hydrolysates produced using HSfla, YSalc, and YSbro exhibited comparatively lower foaming capacities. These variations likely reflect differences in peptide size distribution and the exposure of hydrophobic residues, which are critical for rapid adsorption and film formation at the air–water interface [30]. The FC values obtained in this study are comparable to those reported for yellowstripe scad (*Selaroides leptolepis*) hydrolysates at a similar degree of hydrolysis [38].

Foam stability measured after 10 min followed a similar pattern. The YSalc hydrolysate demonstrated the highest FS, while the lowest stability values were recorded for bromelain-derived hydrolysates (HSbro and YSbro). Enzyme type had a significant effect on FS within each dam (*p* < 0.05); however, no significant differences were observed between hydrolysates from Hirfanlı and Yamula Dam Lakes (*p* > 0.05). These results are consistent with previous findings indicating that the degree of hydrolysis and the resulting peptide profiles influence the formation and resilience of interfacial films, thereby governing foam stabilization [41].

#### 3.7.4. Oil Binding Capacity

**Figure 9:**
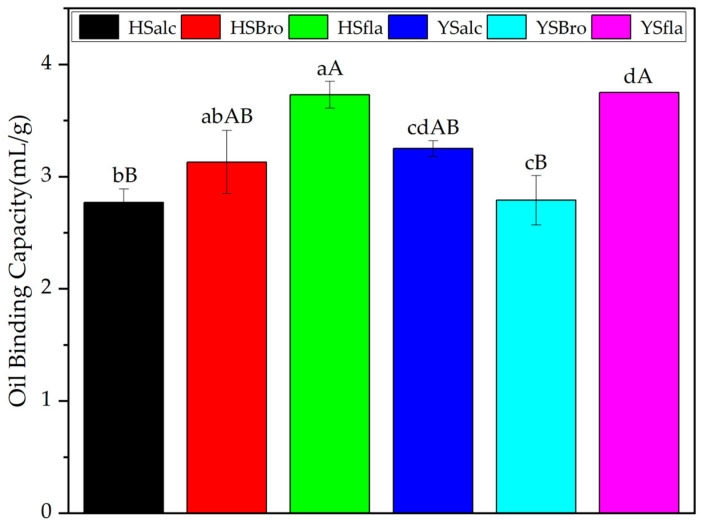
Oil binding capacity of silverfish protein hydrolysates. Results are reported as the mean of three independent measurements with associated standard deviations; error bars represent the corresponding variability (n = 3). Values sharing different lowercase letters differ significantly within each reservoir, whereas different uppercase letters indicate significant differences among all samples (*p* < 0.05). HSalc: Hirfanlı silverfish alcalase hydrolysate, HSbro: Hirfanlı silverfish bromelain hydrolysate, HSfla: Hirfanlı silverfish flavourzyme hydrolysate, YSalc: Yamula silverfish alcalase hydrolysate, YSbro: Yamula silverfish bromelain hydrolysate, YSfla: Yamula silverfish flavourzyme hydrolysate.

Oil binding capacity (OBC) is a key functional attribute of protein hydrolysates, particularly relevant for their application in complex food matrices such as meat products and confectionery systems, where oil retention influences texture, mouthfeel, and product stability [41]. The OBC values of the silverfish protein hydrolysates are shown in Figure 9, ranging from 2.78 ± 0.12 to 3.75 ± 0.12 mL/g. Among the hydrolysates obtained using different enzymes, the sample with the lowest degree of hydrolysis exhibited the highest OBC, supporting the notion that enzyme type and hydrolysis extent strongly influence oil-binding capacity [42]. Statistical analysis revealed no significant differences (*p* > 0.05) between the sample pairs HSbro–YSalc, HSalc–YSbro, and HSfla–YSfla, whereas significant differences (*p* < 0.05) were detected among the remaining sample groups. These observations align with previous research, which reported OBC values of 3.7–7.3 mL/g for herring hydrolysates [43], 2.86–7.07 mL/g for Atlantic salmon hydrolysates [44], and 3.00–3.04 mL/g for anchovy hydrolysates [45]. Overall, the OBC values obtained in this study fall within the typical range (1.0–10.8 mL/g) reported for fish protein hydrolysates [37], confirming their suitability for applications requiring moderate oil retention capacity.

### 3.8. Bioactive Properties of FPH

#### 3.8.1. DPPH Radical Scavenging Activity

**Figure 10:**
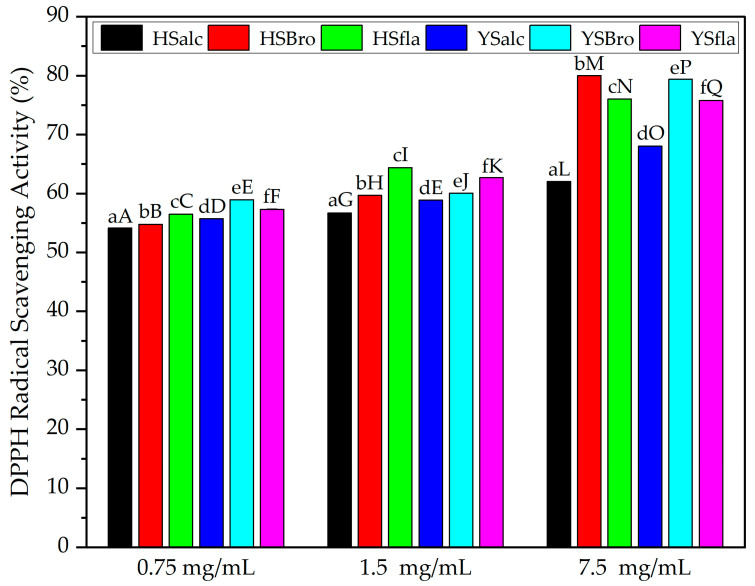
DPPH radical scavenging activity of silverfish protein hydrolysates. Data are expressed as mean ± standard deviation of triplicate determinations. Means with different lowercase letters indicate statistically significant differences (*p* < 0.05) within the same concentration, while different uppercase letters indicate statistically significant differences in all samples (*p* < 0.05). HSalc: Hirfanlı silverfish alcalase hydrolysate, HSbro: Hirfanlı silverfish bromelain hydrolysate, HSfla: Hirfanlı silverfish flavourzyme hydrolysate, YSalc: Yamula silverfish alcalase hydrolysate, YSbro: Yamula silverfish bromelain hydrolysate, YSfla: Yamula silverfish flavourzyme hydrolysate.

The DPPH assay was applied as a rapid screening method to evaluate the radical scavenging capacity of the obtained protein hydrolysates. Antioxidants are compounds that inhibit or delaying oxidative reactions by neutralizing free radicals, which are associated with the development of chronic diseases such as cardiovascular disorders and cancer [46]. Fish protein hydrolysates exhibit inherent antioxidant activity derived from their peptide composition, acting via both free radical scavenging and transition metal chelation mechanisms [47]. The DPPH radical scavenging activities of the hydrolysates produced using different enzymes are presented in Figure 10. At all tested concentrations (0.75, 1.5, and 7.5 mg/mL), the lowest DPPH scavenging activity was recorded in the hydrolysates produced using Alcalase, with values of 54.15%, 56.68%, and 62.04%, respectively. In contrast, bromelain hydrolysates exhibited the highest scavenging activity, reaching 79.99% at 7.5 mg/mL. Flavourzyme-treated samples also showed relatively higher activity compared with Alcalase-treated samples. Hydrolysates with higher DH generally displayed enhanced DPPH scavenging activity, supporting the established mechanism whereby extensive peptide cleavage increases the availability of low-molecular-weight peptides with greater hydrogen- or electron-donating capacity [47]. Conversely, samples hydrolyzed by flavourzyme, which produced the lowest DH, exhibited intermediate antioxidant activity, whereas Alcalase samples—despite having a higher DH than flavourzyme—showed the weakest DPPH response, suggesting that antioxidant capacity is influenced not only by DH but also by enzyme-specific cleavage patterns. This interpretation is further supported by the amino acid composition of the hydrolysates [8]. Bromelain-treated samples contained higher levels of hydrophobic and aromatic amino acids such as leucine, valine, phenylalanine, tyrosine, and tryptophan, all of which are known to enhance radical scavenging through interactions with hydrophobic radicals and stabilization of radical intermediates. In contrast, samples derived from Alcalase and Flavourzyme contained lower proportions of these key antioxidant residues, reflecting their relatively weaker scavenging performance. These findings are partially consistent with Je et al. [48], who evaluated tuna backbone hydrolysates produced with six different enzymes and reported that Alcalase hydrolysates exhibited a notably low scavenging activity of 4.82% at 1.5 mg/mL. In the present study, silverfish Alcalase hydrolysates demonstrated an average activity of approximately 57% at the same concentration, corresponding to an approximately 11-fold increase. This discrepancy may be attributed to differences in raw material composition, peptide profiles, and especially hydrolysis duration, which plays a decisive role in antioxidant capacity [41]. Prolonged hydrolysis can generate very small peptides or free amino acids that have reduced DPPH scavenging activity [38]. Overall, the obtained results indicate that enzyme type strongly influences radical scavenging activity, with Bromelain and Flavourzyme producing peptides with superior antioxidant potential compared with Alcalase. The results demonstrate that the antioxidant potential of silverfish protein hydrolysates is shaped by a combination of enzyme specificity, extent of hydrolysis, and resulting peptide structural features, with bromelain emerging as the most effective enzyme for generating peptides with strong radical scavenging activity.

#### 3.8.2. Determination of Antimicrobial Activity

The antimicrobial activities of silverfish protein hydrolysates obtained from Hirfanlı (HS) and Yamula (YS) reservoirs using three proteolytic enzymes—Alcalase (alc), Bromelain (bro), and Flavourzyme (fla)—were evaluated against *E. coli, B. cereus, Staph. aureus,* and *S. typhimurium* (Table 3). The inhibition zone diameters (mm) are measured at three concentrations (1:1, 1:2, and 1:4 μg/mL). The antimicrobial activity of silverfish protein hydrolysates is due to the release of antimicrobial peptides during enzymatic hydrolysis. Antimicrobial peptides act against bacteria by disrupting their DNA, RNA, and protein synthesis or by interacting with and forming pores in the bacterial cell membrane [49]. Enzymes break down protein chains at various sites, producing a diverse range of peptides with different sequences, molecular weights, and structures [50]. Ultimately, this variation in peptide composition may lead to differing levels of antimicrobial effectiveness of silverfish protein hydrolysates prepared with different enzymes against various bacterial species. All hydrolysates exhibited antimicrobial activity, showing a clear dose-dependent reduction in the size of the inhibition zone with dilution. Against *E. coli*, the strongest inhibition was recorded in the flavourzyme-treated hydrolysates (HSfla and YSfla). The pronounced activity of Flavourzyme hydrolysates is consistent with previous findings reporting high anti-*E. coli* activity in fish-derived peptide fractions such as those from turbot [51]. A progressive decline in inhibition zone diameter was observed with decreasing hydrolysate concentration, confirming concentration-dependent antimicrobial behavior across all samples. For *B. cereus*, the highest antimicrobial effect was observed in HSalc, whereas YSbro exhibited the strongest inhibition among Yamula samples. These variations emphasize the influence of both enzyme specificity and raw material origin on the antimicrobial performance of the hydrolysates. Inhibition of *Staph. aureus* was comparatively lower across all enzymes–location combinations, suggesting that this organism exhibits higher resistance. Nevertheless, YSbro and HSfla demonstrated relatively greater inhibition than other hydrolysates, consistent with reports of moderate anti-*Staph. aureus* activity in marine protein hydrolysates [52]. Gram-negative bacteria have an additional outer membrane composed of lipopolysaccharides, which makes them more resistant than Gram-positive bacteria [52]. However, in this study, the low activity observed against Staph. aureus may be due to the specific peptide composition of the silverfish protein hydrolysate. This is because peptides produced by different enzymes can influence the susceptibility of each bacterial group to varying degrees. Understanding these differences is important for identifying potential uses, such as targeted antimicrobial solutions for food preservation or aquaculture. This supports the development of safer and more sustainable food and pharmaceutical products. Against *S. typhimurium*, both HSalc and YSalc exhibited stronger antimicrobial activity than bro- and fla-derived hydrolysates. This suggests that Alcalase treatment generated peptide fractions with enhanced bioactivity toward Gram-negative species. Overall, Alcalase- and Flavourzyme-treated hydrolysates exhibited more pronounced antimicrobial properties than those produced with Bromelain. The maximum inhibition zone recorded in this study was 33.5 mm for HSalc against *B. cereus*, whereas the minimum, 18.0 mm, was observed for YSalc against *Staph. aureus*. These findings demonstrate that both the enzyme type and the reservoir origin significantly modulate the antimicrobial activity of silverfish hydrolysates. The results collectively highlight that peptide composition driven by enzyme specificity plays a crucial role in determining antimicrobial potency [41].

#### 3.8.3. Angiotensin-I Converting Enzyme (ACE) Inhibitory Activity

The ACE-inhibitory properties of the hydrolysates differed significantly as a function of enzyme type and sampling location. Within the Hirfanlı group, the strongest inhibition was observed for HSfla (21.45%), followed by HSbro (20.74%) and HSalc (18.75%, *p* < 0.05). A comparable trend was observed in Yamula samples: YSfla (22.96%) exhibited the highest activity (*p* < 0.05), slightly exceeding YSalc (22.77%) and YSbro (22.35%). Across all treatments, Yamula-derived hydrolysates consistently demonstrated higher inhibition than their Hirfanlı counterparts, suggesting that site-specific raw material characteristics may favor the release of ACE-active peptide sequences. The superior performance of Flavourzyme in both reservoirs is likely attributed to its combined endo- and exopeptidase activities, which facilitate the generation of short, hydrophobic peptides known to interact effectively with the active site of ACE. Such peptides typically display high binding affinity toward the catalytic pocket, thereby enhancing inhibitory capacity. Recent literature supports the activity range observed in this study. Sapatinha et al. [53] reported ACE-inhibitory activities of 64.65–78.12% at 5 mg/mL for hydrolysates produced from frozen fish processing co-products. Similarly, Elavarasan and Shamasundar [54] documented 43–71% inhibition at 5 mg/mL for hydrolysates derived from freshwater carp. Hu et al. [55] demonstrated that monkfish hydrolysates produced using sequential Alcalase–Neutrase digestion exhibited 53.22% inhibition at 2.5 mg/mL. The ACE-inhibitory activities achieved in the present work at 1 mg/mL therefore fall within the broad inhibitory range previously reported for fish protein hydrolysates, despite having been assessed at a substantially lower concentration. Overall, these findings confirm that *A. boyeri* hydrolysates possess notable ACE-inhibitory potential, underscoring their relevance as natural antihypertensive ingredients. These enzyme-dependent differences further underscore the pivotal role of enzyme specificity in shaping the generation of physiologically relevant bioactive peptides.

## 4. Conclusions

This study demonstrated that the invasive fish *A. boyeri* can be effectively valorized as a sustainable source of protein hydrolysates with desirable functional and bioactive properties. The hydrolysates produced using Alcalase, Bromelain, and Flavourzyme displayed distinct techno-functional characteristics governed primarily by enzyme specificity, the extent of hydrolysis, and peptide composition. Bromelain treatment produced hydrolysates with the highest degree of hydrolysis, which was reflected by enhanced antioxidant activity, particularly in DPPH radical-scavenging assays. Flavourzyme-derived hydrolysates exhibited superior emulsifying performance, whereas Alcalase treatments generally produced higher foaming capacity and stability. Variations in zeta potential across pH levels further revealed the influence of hydrolysis on colloidal behavior, supporting the observed differences in emulsification and foaming properties. The oil-binding capacities of the hydrolysates were within the expected range for marine proteins and remained consistent with values reported for other commercially relevant species. From an application perspective, the pronounced foaming properties of the hydrolysates support their potential use in aerated food products, whereas their emulsifying performance indicates suitability as natural emulsifiers in oil-in-water systems. The consistently high protein solubility further broadens their applicability to liquid and protein-fortified formulations. Collectively, these functional attributes highlight the feasibility of utilizing *A. boyeri* hydrolysates as functional ingredients in diverse food matrices. Beyond functional attributes, the hydrolysates demonstrated antimicrobial activity against foodborne pathogens including *E. coli*, *B. cereus*, *Staph. aureus*, and *S. typhimurium*. Enzyme-specific differences were evident, with Alcalase and Flavourzyme generally yielding peptides with stronger inhibitory effects. Moreover, all hydrolysates exhibited angiotensin-converting enzyme (ACE) inhibitory activity at appreciable levels, with flavourzyme-treated samples showing the highest inhibition, highlighting their potential relevance for antihypertensive applications and expanding the biofunctional profile of *A. boyeri* peptides. The observed antimicrobial and antioxidant activities, together with ACE-inhibitory activity, underscore the potential of *A. boyeri* hydrolysates as multifunctional ingredients suitable for incorporation into food formulations that require both preservation and functional enhancement. Overall, the findings support the suitability of *A. boyeri* as a high-value raw material for the production of bioactive protein hydrolysates, contributing to the circular bioeconomy and to invasive species management strategies. Despite these promising findings, this study has certain limitations. The bioactivities were evaluated using unfractionated hydrolysates and in vitro assays, and peptide purification and in vivo validation were not conducted. Further studies addressing peptide isolation, structure–activity relationships, and performance in real food systems will be necessary to fully establish the application potential of *A. boyeri* protein hydrolysates. Future research should focus on (i) isolation and structural characterization of individual bioactive peptides, (ii) evaluation of in vivo antioxidant, antimicrobial, and antihypertensive efficacy, (iii) incorporation of hydrolysates into model food systems to assess sensory and physicochemical stability, and (iv) optimization of enzymatic hydrolysis at pilot and industrial scales. Such efforts will further clarify the applicability of silverfish-derived hydrolysates as natural, functional ingredients for clean-label and health-oriented food products.

## Figures and Tables

**Table 1:** Amino acid composition of invasive silverfish (*Atherina boyeri*) protein hydrolysates (mg/100 g).

Amino Acid	HSalc	HSbro	HSfla	YSalc	YSbro	YSfla
Aspartic acid	5520.5 ± 1.24	5712 ± 0.30	6175.5 ± 0.81	5410 ± 0.58	7035 ± 1.13	5826.5 ± 1.40
Glutamic acid	9596 ± 0.68	10,819 ± 0.11	11,230 ± 0.59	9477 ± 0.61	11,526.5 ± 0.67	10,745 ± 0.88
Serine	3335.5 ± 0.02	3656 ± 0.06	3676.5 ± 0.10	3412.5 ± 0.10	4184 ± 0.30	3649 ± 0.16
Glycine	3636.5 ± 0.06	4121 ± 0.24	4360.5 ± 0.79	3515.5 ± 0.18	4788.5 ± 0.34	4502 ± 0.35
Histidine	2736.5 ± 0.13	2817 ± 0.10	2833.5 ± 0.37	2567.5 ± 0.91	2996.5 ± 0.64	2690.5 ± 0.45
Arginine	3911 ± 0.43	4469 ± 0.19	4075 ± 1.87	3939.5 ± 0.31	4091 ± 0.83	4265 ± 1.76
Threonine	4441.5 ± 0.18	4916 ± 1.08	4299 ± 0.26	3940 ± 0.86	5116.5 ± 0.37	4134 ± 4.82
Alanine	3813.5 ± 0.32	4365 ± 0.60	4270 ± 0.33	3409.5 ± 0.89	4593.5 ± 0.38	3988.5 ± 1.58
Proline	3037.5 ± 0.49	3828 ± 1.92	3211.5 ± 0.64	2774.5 ± 0.79	3737 ± 0.61	3275 ± 2.50
Tyrosine	2235.5 ± 0.03	2075 ± 1.36	1541 ± 1.19	1669 ± 0.34	2701 ± 0.21	1599 ± 0.18
Valine	3494.5 ± 0.59	3911 ± 0.43	3474.5 ± 0.67	2968 ± 0.57	3921.5 ± 0.56	3120 ± 0.54
Methionine	1786.5 ± 0.04	2246 ± 0.69	1749.5 ± 0.12	1434 ± 0.10	2135.5 ± 0.43	1499 ± 0.38
Isoleucine	3220.5 ± 0.11	3539 ± 1.34	3156 ± 0.09	2692 ± 0.05	3416 ± 0.46	2931.5 ± 0.17
Leucine	5266 ± 0.56	6152 ± 0.17	5392 ± 1.15	4513 ± 0.09	5788 ± 0.86	4927 ± 0.14
Phenylalanine	2844.5 ± 0.37	3415 ± 0.50	2835 ± 0.30	2373 ± 0.42	2745.5 ± 1.00	2398 ± 0.29
Lysine	10,872 ± 0.46	10,776 ± 0.95	11,044 ± 0.61	11,802 ± 0.43	11,072 ± 0.70	12,207.5 ± 0.38
Total	69,748 ± 0.16	76,814 ± 0.09	73,323.5 ± 0.11	65,897 ± 0.21	79,848 ± 0.17	71,757.5 ± 0.77

Data are expressed as mean ± standard deviation (n = 3) from triplicate determinations. HSalc: Hirfanlı silverfish alcalase hydrolysate, HSbro: Hirfanlı silverfish bromelain hydrolysate, HSfla: Hirfanlı silverfish flavourzyme hydrolysate, YSalc: Yamula silverfish alcalase hydrolysate, YSbro: Yamula silverfish bromelain hydrolysate, YSfla: Yamula silverfish flavourzyme hydrolysate.

**Table 2:** Color properties of silverfish protein hydrolysates.

Samples	L*	a*	b*
HSalc	97.17 ± 0.78 ^aAB^	−1.67 ± 0.16 ^aAB^	29.46 ± 0.38 ^aA^
HSbro	97.02 ± 1.32 ^aAB^	−1.40 ± 0.08 ^aA^	29.38 ± 0.28 ^aAB^
HSfla	95.53 ± 0.40 ^aB^	−1.50 ± 0.12 ^aAB^	25.44 ± 0.74 ^bC^
YSalc	97.87 ± 0.19 ^bAB^	−2.03 ± 0.04 ^bC^	28.24 ± 1.23 ^cAB^
YSbro	98.90 ± 0.82 ^bA^	−1.75 ± 0.03 ^cBC^	28.54 ± 0.13 ^cAB^
YSfla	97.81 ± 1.19 ^bAB^	−1.42 ± 0.11 ^dA^	27.57 ± 0.56 ^cB^

Data are expressed as mean ± standard deviation (n = 3) of triplicate determinations. Values sharing different lowercase letters differ significantly within each reservoir, whereas different uppercase letters indicate significant differences among all samples (*p* < 0.05). HSalc: Hirfanlı silverfish alcalase hydrolysate, HSbro: Hirfanlı silverfish bromelain hydrolysate, HSfla: Hirfanlı silverfish flavourzyme hydrolysate, YSalc: Yamula silverfish alcalase hydrolysate, YSbro: Yamula silverfish bromelain hydrolysate, YSfla: Yamula silverfish flavourzyme hydrolysate.

**Table 3:** Antimicrobial activity of silverfish protein hydrolysates at different concentrations against pathogenic bacteria.

Samples	Concentration (µg/mL)	*E. coli*	*B. cereus*	*Staph. aureus*	*S. typhimurium*
HSalc	1:1	23.0 ± 1.4	33.5 ± 2.1	21.5 ± 0.7	30.0 ± 1.4
	1:2	n.d.	29.0 ± 1.4	n.d.	24.5 ± 2.1
	1:4	n.d.	23.0 ± 1.4	n.d.	23.0 ± 1.4
HSbro	1:1	28.5 ± 0.7	30.5 ± 0.7	22.0 ± 1.4	28.5 ± 0.7
	1:2	24.0 ± 1.4	27.0 ± 0.0	n.d.	22.5 ± 0.7
	1:4	23.0 ± 1.4	24.0 ± 2.8	n.d.	20.0 ± 1.4
HSfla	1:1	30.5 ± 0.7	30.5 ± 0.7	24.0 ± 0.0	28.5 ± 2.1
	1:2	26.0 ± 2.8	24.5 ± 0.7	n.d.	22.0 ± 1.4
	1:4	n.d.	24.0 ± 0.0	n.d.	19.0 ± 1.4
YSalc	1:1	25.0 ± 0.0	28.0 ± 1.4	18.0 ± 1.4	29.0 ± 1.4
	1:2	23.0 ± 1.4	26.5 ± 0.7	n.d.	25.0 ± 1.4
	1:4	n.d.	22.5 ± 0.7	n.d.	19.0 ± 1.4
YSbro	1:1	30.0 ± 1.4	29.5 ± 0.7	24.5 ± 0.7	25.5 ± 0.7
	1:2	29.5 ± 0.7	25.5 ± 0.7	n.d.	22.5 ± 0.7
	1:4	24.5 ± 0.7	22.0 ± 1.4	n.d.	18.0 ± 1.4
YSfla	1:1	31.0 ± 1.4	27.5 ± 0.7	20.5 ± 0.7	26.5 ± 0.7
	1:2	29.5 ± 0.7	22.5 ± 0.7	n.d.	23.5 ± 0.7
	1:4	27.5 ± 0.70	22.5 ± 0.70	n.d.	20.0 ± 1.41

Data are expressed as mean ± standard deviation (*n* = 3) of triplicate determinations. HSalc: Hirfanlı silverfish alcalase hydrolysate, HSbro: Hirfanlı silverfish bromelain hydrolysate, HSfla: Hirfanlı silverfish flavourzyme hydrolysate, YSalc: Yamula silverfish alcalase hydrolysate, YSbro: Yamula silverfish bromelain hydrolysate, YSfla: Yamula silverfish flavourzyme hydrolysate. n.d.: Not determined.

## Data Availability

The original contributions presented in this study are included in the article. Further inquiries can be directed to the corresponding authors.

## Abbreviations

The following abbreviations are used in this manuscript:

HSalc	Hirfanlı silverfish alcalase hydrolysate
HSbro	Hirfanlı silverfish bromelain hydrolysate
HSfla	Hirfanlı silverfish flavourzyme hydrolysate
YSalc	Yamula silverfish alcalase hydrolysate
YSbro	Yamula silverfish bromelain hydrolysate
YSfla	Yamula silverfish flavourzyme hydrolysate
OBC	Oil binding capacity
EAI	Emulsion activity index
ESI	Emulsion stability index
PBS	Phosphate-buffered saline
SDS	Sodium dodecyl sulfate
TUIK	Turkish Statistical Institute
FC	Foam Capacity
FS	Foam Stability
